# Schizophrenia likely related to be with cadasil: A case report

**DOI:** 10.1192/j.eurpsy.2021.2141

**Published:** 2021-08-13

**Authors:** D. Göy, Ö. Şahmelikoğlu Onur, U. Yesilkaya

**Affiliations:** Psychiatry, Bakirkoy Research & Training Hospital for Psychiatry, Neurology and Neurosurgery, İstanbul, Turkey

**Keywords:** CADASIL syndrome, schizophrénia, organic psychosis

## Abstract

**Introduction:**

CADASIL (Cerebral Autosomal Dominant Arteriopathy with Subcortical Infarcts and Leukoencephalopathy) is an inherited disease caused by mutations in the Notch3 gene in chromosome 19.The clinical features are primarily neurological, which include recurrent transient ischaemic attacks, strokes, and migraines.However, psychiatric manifestations such as severe depression, psychosis, changes in behavior and personality have also been reported in CADASIL. Symptoms and disease onset vary widely, with signs typically appearing in the mid-30s.Some individuals may not show signs of the disease until later in life.

**Objectives:**

In this case report, we present a possible case of CADASIL, a 67-year old female patient diagnosed with schizophrenia fifteen years ago to discuss the co-occurrence of these conditions in the light of the literature.

**Methods:**

Hospitalization records of the patient viewed.

**Results:**

Our patient suffered from sleep disturbances, hearing religious voices and in psychiatric examination resistant to treatment psychotic symptoms such as blunted affect, mystic, persecutory delusions, auditory hallucinations were present.Cranial magnetic resonance imaging scan was performed and revealed leukoencephalopathy, high-signal intensity lesions in the periventricular white matter consistent with a diagnosis of CADASIL.Atypical antipsychotics found to be effective in treating psychotic symptoms in our case.

**Conclusions:**

Persistent psychotic symptoms despite adequate antipsychotic treatment in patients diagnosed with schizophrenia, also with pathological findings in MRI an organic cause such as CADASIL must be considered. Further studies are needed to better understand the exact impacts of cerebral tissue lesions and psychiatric symptoms in CADASIL patients.

**Disclosure:**

No significant relationships.

